# Diagnostic Performance of a Silver-Amplified vs. a Non-Amplified Lateral Flow Kit for Adenoviral Conjunctivitis: A Multicenter Prospective Study

**DOI:** 10.3390/v17111442

**Published:** 2025-10-29

**Authors:** Tsuguto Fujimoto, Nozomu Hanaoka, Kenichiro Takahashi, Hisatoshi Kaneko, Masaaki Kobayashi, Hisashi Nakagawa, Hiroshi Hatano, Tomoko Tsukahara-Kawamura, Hironori Migita, Kentaro Nakamura, Kiyoharu Kuramoto, Eiichi Uchio

**Affiliations:** 1 Department of Fungal Infection, National Institute of Infectious Diseases, Japan Institute for Health Security, Tokyo 162-8640, Japan; 2 Center for Emergency Preparedness and Response, National Institute of Infectious Diseases, Tokyo 162-8640, Japan; nozomu@niid.go.jp (N.H.); takaken@niid.go.jp (K.T.); 3 Hobara Eye Clinic, Date 960-0612, Japan; h-kane@chive.ocn.ne.jp; 4 Kobayashi Pediatric Clinic, Fujieda 426-0067, Japan; koba-m@if-n.ne.jp; 5 Tokushima Eye Clinic, Tokyo 189-0024, Japan; naka-h@muh.biglobe.ne.jp; 6 Hatano Eye Clinic, Fujisawa 251-0052, Japan; hrshhtn@gmail.com; 7 Department of Ophthalmology, Fukuoka University School of Medicine, Fukuoka 814-0180, Japan; ttsukahara@adm.fukuoka-u.ac.jp (T.T.-K.); uchio-eiichi-ff@ihwg.jp (E.U.); 8 Migita Eye Clinic, Chikushino 818-0083, Japan; migita-ganka@lilac.ocn.ne.jp; 9 FUJIFILM Corporation, Tokyo 107-0052, Japan; kentaro.nakamura@fujifilm.com (K.N.); kiyoharu.kuramoto@fujifilm.com (K.K.)

**Keywords:** adenoviral conjunctivitis, lateral flow assay, diagnostic accuracy, epidemic keratoconjunctivitis

## Abstract

Accurate diagnosis of adenoviral conjunctivitis is critical for timely treatment and infection control. However, conventional lateral flow kits often lack sufficient sensitivity, especially in mild cases. This multicenter prospective study evaluated the diagnostic performance of silver-amplified lateral flow kits (SA-LFKs) compared with non-silver-amplified kits (NSA-LFKs), using real-time polymerase chain reaction (qPCR) as the reference standard. Tear samples from 200 patients with suspected adenoviral conjunctivitis were collected across four clinics and analyzed for sensitivity, specificity, and genotype coverage. The SA-LFK demonstrated significantly higher sensitivity (86.0%) than the NSA-LFK (72.0%) (*p* < 0.001), with both kits showing high specificity (100% and 98.1%, respectively). Pooled analysis revealed that sensitivity was significantly lower in mild-to-moderate cases than in severe cases for both kits, suggesting that clinical severity influences detection performance. Across all severity levels, the SA-LFK consistently demonstrated higher sensitivity than the NSA-LFK, including in mild-to-moderate cases (77.8% vs. 59.3%, *p* = 0.004), supporting its superior diagnostic performance. The SA-LFK showed robust performance across eight identified adenovirus genotypes and maintained higher positivity rates even at lower viral loads. These findings support the clinical utility of SA-LFKs for early diagnosis and outbreak containment in diverse settings.

## 1. Introduction

Adenoviruses (AdVs) are common pathogens responsible for epidemic keratoconjunctivitis (EKC) and pharyngoconjunctival fever (PCF), both of which are highly contagious ocular infections [1]. Owing to their ability to remain infectious in the environment for extended periods, AdVs pose a significant risk for nosocomial transmission, institutional outbreaks, and community-wide epidemics [2,3].

Accurate and timely diagnosis is essential for preventing the spread of AdV infections, as even mild or inapparent human adenoviral conjunctival infections can play a role in the spread of nosocomial infections [4]. While lateral flow (LF) kits are widely used for point-of-care testing [2], their diagnostic sensitivity, particularly for ocular specimens, has not been sufficiently recognized as a concern among ophthalmologists. This may be attributable to the limited availability of comparative data on different LF technologies. In clinical practice, non-silver-amplified LF kits (NSA-LFKs) are more commonly used than silver-amplified LF kits (SA-LFKs), owing to the lack of clinical studies evaluating these methods using tear fluid. However, this preference may contribute to underdiagnosis, as NSA-LFKs generally exhibit lower sensitivity, potentially leading to false-negative results.

In recent years, tear fluid has increasingly supplanted conjunctival swab specimens as the preferred sample type for adenoviral ocular diagnostics in Japan. Despite this shift, the lack of shared performance data has hindered the broader recognition of the diagnostic limitations associated with NSA-LFKs.

SA-LFKs employ a chemical amplification step that enlarges metal nanoparticles bound to antigen–antibody complexes on the test line, thereby increasing optical density and improving analytical sensitivity. In contrast, NSA-LFKs rely solely on native particle signal without amplification. This difference may influence diagnostic performance, particularly when antigen levels are low.

We have previously demonstrated that SA-LFKs can achieve 10- to 250-fold higher sensitivity than NSA-LFKs, based on evaluations conducted under laboratory conditions [5]. Furthermore, in a single-clinic study conducted during an outbreak of AdV 54, SA-LFKs exhibited a sensitivity of 98.3% when compared with polymerase chain reaction (PCR) [6]. However, this study was limited to a single institution and a short observation period, prompting the need for a multicenter clinical evaluation using patient-derived samples to validate the real-world diagnostic performance of SA-LFKs.

To address these limitations, we aimed to compare the diagnostic performance of SA-LFKs and NSA-LFKs over a two-year period across multiple clinical sites in different regions of Japan, using a diverse set of AdV types. Recognizing that diagnostic metrics may vary based on the reference standard and that accurate typing of AdV requires not only the commonly used hexon region but also the penton and fiber regions [7,8,9], four different PCR techniques—quantitative real-time PCR (qPCR) [5], hexon-nested PCR [10], penton PCR [11], and fiber multiplex PCR [12]—were used to comprehensively evaluate the sensitivity and specificity of both LF approaches. Additionally, diagnostic performance was stratified by clinical severity to assess the practical utility of SA-LFKs in real-world settings.

This study provides robust clinical evidence supporting the superior diagnostic sensitivity of SA-LFKs, thereby informing future guidelines for adenoviral ocular diagnostics.

## 2. Materials and Methods

### 2.1. Patients

This prospective study included 200 patients who visited one of four geographically distinct medical institutions [13] located in Fukushima, Shizuoka, Tokyo, and Kanagawa prefectures between 19 May 2018, and 24 January 2020. These included three ophthalmology clinics and one pediatric clinic. All patients were clinically suspected by their attending physicians to have adenoviral conjunctivitis, with follicular conjunctival lesions observed.

At the time of specimen collection, the severity of conjunctivitis was assessed and documented by the attending ophthalmologist based on the anatomical extent of inflammation in the palpebral conjunctiva. Severity was classified as mild when inflammation was limited to the lower palpebral conjunctiva; moderate when inflammation involved both upper and lower palpebral conjunctiva, without extension to the conjunctival fornix; and severe when inflammation involved both upper and lower palpebral conjunctiva, extending to the conjunctival fornix. These classifications, based on previous studies [1,14,15], were subsequently used to evaluate the diagnostic performance of LF kits in relation to clinical severity.

The study was conducted in accordance with the Declaration of Helsinki. The study protocol, including specimen collection and testing, was approved by the Ethics Committee of the National Institute of Infectious Diseases (Approval No. 1088, approved on 10 February 2020) and by the Life Science Ethics Review Committee of FUJIFILM Corporation (Clinical Research #074-1, approved on 19 January 2018).

### 2.2. Blinding and Operational Details

Clinical severity grading was performed before LF testing. SA-LFK and NSA-LFK testing was performed in a blinded research setting separate from clinical decision-making.

### 2.3. Clinical Specimens

Clinical specimens were collected from all 200 patients with suspected AdV conjunctivitis and follicular lesions, after obtaining written informed consent. Tear fluid, including conjunctival exudate, was collected from the conjunctiva using a conjunctival exudate collection device included in the kit, as previously described [6]. Specifically, approximately 25 μL of tear fluid was obtained by gently pressing the strip against the conjunctiva for 5 s.

### 2.4. LF Testing

Two LF kits were used:

Amplification method: FUJI DRI-CHEM IMMUNO AG Cartridge Adeno OPH (Fujifilm Corporation, Tokyo, Japan) [5]. This LF kit is also sold by ROHTONITTEN Co., Ltd. (Nagoya, Japan) as “Quick Chaser Auto Adeno Eye,” which was not used in this study.

Non-amplification method: FUJI DRI-CHEM IMMUNO AG Cartridge Adeno (Fujifilm Corporation, Tokyo, Japan) [6].

Approximately 25 μL of tear fluid was collected using a conjunctival exudate collection device and dispersed into 600 μL of the extraction buffer provided with each kit. Four drops (approximately 150 μL) of the mixture were then applied to each LF kit. Results were interpreted using the FUJI DRI-CHEM IMMUNO AG1 automated reader, rather than by visual inspection. This reader is sold as “Quick Chaser Immuno Reader” by ROHTONITTEN Co., Ltd. and MIZUHO MEDY Co., Ltd. (Saga, Japan).

The LF kit results were subsequently compared with recorded clinical severity to assess the sensitivity of each kit across different severity levels.

### 2.5. AdV DNA Extraction

Viral DNA was extracted from 200 μL of the residual extraction buffer used in the LF test. The High Pure Viral Nucleic Acid Kit (Roche Diagnostics, Mannheim, Germany) was used, and DNA was eluted in 50 μL of water for PCR analysis.

### 2.6. PCR Systems and Sequencing

qPCR targeting the hexon C4 region was employed as the gold standard for AdV detection, based on a previously reported method [5,16], with modifications to the primers and thermal conditions for type 54 [5].

To ensure accurate interpretation of SYBR Green-based qPCR results, a cutoff value of 4.6 copies/µL was used, determined from standard curve and melt-curve analyses. Samples below this threshold were considered negative to minimize false positives due to background fluorescence or primer-dimer artifacts.

In addition to qPCR, three conventional PCR methods were used for AdV genotyping: nested PCR targeting the hexon region [10], PCR targeting the penton region [11], and PCR targeting the fiber region [12]. These methods were applied to samples that tested positive using qPCR to determine the AdV type.

### 2.7. AdV Typing

AdV types were determined based on sequences obtained from the abovementioned PCR methods, following previously reported methods. The NCBI BLAST tool (https://blast.ncbi.nlm.nih.gov/Blast.cgi, accessed on 26 October 2025) was utilized for similarity searches. Reference data and classification information were obtained from the Human Adenovirus Working Group website (http://hadvwg.gmu.edu/, accessed on 26 October 2025) to support the identification and characterization of AdV sequences. The accession numbers for the reference AdVs used for typing are listed in Supplementary Table S1.

### 2.8. Statistical Analysis

Diagnostic accuracy metrics, including sensitivity, specificity, positive predictive value (PPV), and negative predictive value (NPV), were calculated with 95% confidence intervals (95% CI) using the Wilson method. Paired comparisons of sensitivity and specificity between kits were performed using McNemar’s test.

To assess whether viral load differed among severity groups, adenoviral DNA copy numbers quantified by qPCR were compared using the Kruskal–Wallis test. Post hoc pairwise comparisons were conducted using Dunn’s multiple comparison test. Severity groups were defined as mild (*n* = 14), moderate (*n* = 109), and severe (*n* = 77), based on the anatomical extent of conjunctival inflammation. Due to the limited number of mild cases, mild and moderate groups were pooled for statistical comparison with the severe group in certain analyses. Between-group comparisons of sensitivity by severity were assessed using McNemar’s test.

Receiver operating characteristic (ROC) curve and area under the curve (AUC) analyses were not applicable because the automated reader provided binary outputs only. Statistical significance was set at *p* < 0.05. Statistical computations were performed using GraphPad Prism 10.5.0 and QuickCalcs (GraphPad Software, San Diego, CA, USA).

## 3. Results

### 3.1. Diagnostic Performance of Lateral Flow Kits Compared with qPCR

The diagnostic performance of two LF kits (SA-LFK and NSA-LFK) was evaluated in a prospective cohort of 200 patients with clinically suspected adenoviral conjunctivitis, using qPCR as the reference standard.

A comprehensive summary of individual case data, including clinical severity, viral load, and genotyping results, is provided in Supplementary Table S1.

Key diagnostic metrics, including sensitivity, specificity, false-negative rate, false-positive rate, PPV, and NPV, were calculated for both LF kits (Table 1 and Table 2).

However, the SA-LFK showed no false positives, whereas the NSA-LFK showed two false positives against qPCR (specificity 105/107, 98.1%). This trend was consistently observed across all four participating clinical facilities. Notably, even in facilities with limited sample diversity—such as clinic A, which included only PCR-positive specimens (*n* = 10) and clinic B, which included only one PCR-positive sample out of 11—the SA-LFK showed superior performance. In clinic A, the SA-LFK detected nine out of ten positive cases, whereas the NSA-LFK detected only seven. In clinic B, both kits detected a single positive case. Additionally, two cases that were positive only based on the NSA-LFK were negative by qPCR and were therefore considered false positives.

McNemar’s test confirmed the significance of these findings. Among 93 PCR-positive specimens, the SA-LFK showed significantly higher sensitivity than the NSA-LFK (*p* < 0.001). In contrast, analysis of 107 PCR-negative specimens showed no significant difference in specificity between the two kits (*p* = 0.48).

These findings suggest that the diagnostic performance of the SA-LFK was consistently favorable across diverse clinical environments, including facilities with limited or skewed sample distributions, where similar trends were observed.

### 3.2. LF Kit Positivity by Adenovirus Copy Number

**Figure 1:**
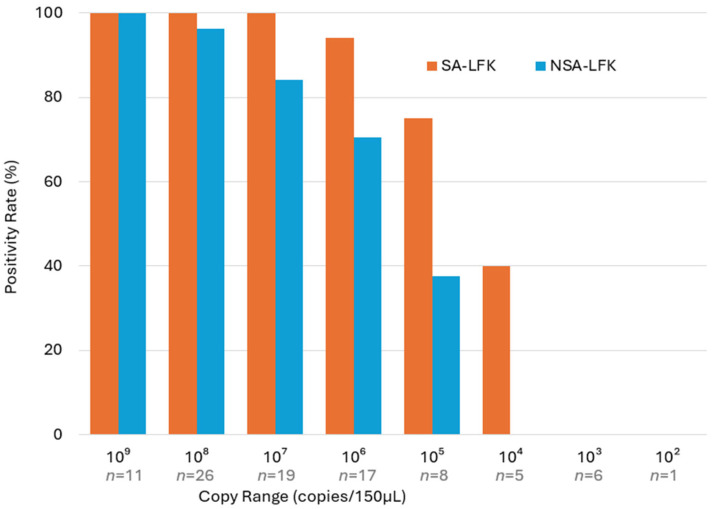
Positivity rates (%) of LF detection by AdV copy number in the extraction buffer.

The relationship between AdV copy number in 150 μL of LF extraction buffer and LF kit results is shown in Figure 1. The SA-LFK detected all samples with copy numbers ranging from1.11 × 10^7^ to 8.19 × 10^9^ (100%, 56/56), whereas the NSA-LFK missed four samples (7.1%). At lower copy numbers (10^4^ to 10^6^), the SA-LFK maintained higher positivity rates than the NSA-LFK. Both kits failed to detect any samples with copy numbers ≤10^3^.

### 3.3. Genotyping by Hexon Nested PCR and LF Kit Positivity

**Figure 2:**
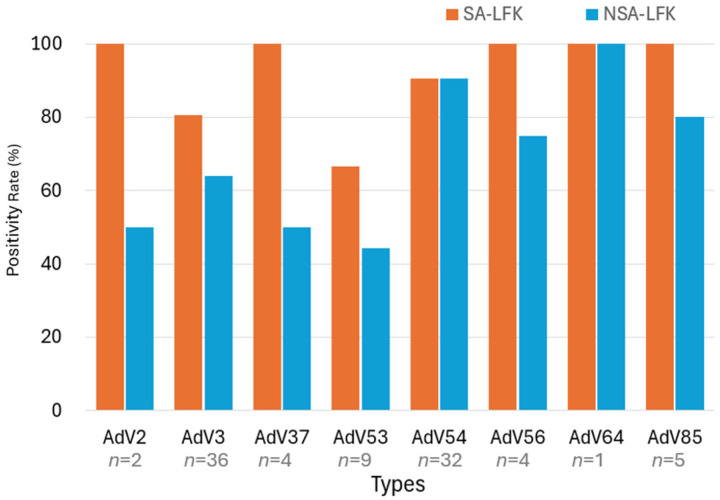
Positivity rates (%) of lateral flow (LF) detection across different AdV genotypes.

Hexon nested PCR [10] was used to genotype AdV in 93 qPCR-positive samples, identifying eight distinct types including types 3, 53, and 54. The SA-LFK showed higher positivity rates across most types than the NSA-LFK. For type 53, the SA-LFK detected six out of nine cases (66.7%), whereas the NSA-LFK detected four of nine cases (44.4%). In contrast, the SA-LFK achieved detection rates exceeding 80% for the majority of other genotypes, highlighting its superior sensitivity across a broad spectrum of adenoviral strains (Figure 2).

### 3.4. Additional PCR Typing Results

Penton and fiber PCR methods were used for qualitative genotyping in qPCR-positive samples. Penton PCR [11] identified AdV species in 54 of 55 samples as species D using hexon nested PCR (98.2%), while fiber multiplex PCR [12] detected AdV in 85 of 93 hexon PCR-positive samples (91.4%). Although these methods provide supportive evidence for genotype identification, they are not used for diagnostic performance evaluation.

A summary of the genotype results, along with the corresponding clinical symptoms and viral loads for each case, is provided in Supplementary Table S1.

### 3.5. Diagnostic Performance of Lateral Flow Kits by Clinical Severity

The diagnostic performance of the SA-LFK and the NSA-LFK was evaluated across three severity levels: mild (*n* = 14), moderate (*n* = 109), and severe (*n* = 77).

The SA-LFK consistently showed higher sensitivity and NPV across all severity categories compared with the NSA-LFK. Specificity and PPV remained high for both kits, with the SA-LFK maintaining 100% across all metrics. Notably, sensitivity and NPV were lowest in mild cases for both kits, reflecting the challenge of detection in less severe cases. The SA-LFK showed relatively better performance, even in mild cases.

Due to the limited number of mild cases (*n* = 14), an additional pooled analysis combining mild and moderate cases was conducted to improve statistical reliability (Table 3, Mild + Moderate). In this pooled group, the SA-LFK demonstrated a sensitivity of 77.8% (95% CI: 65.1–86.8) and a specificity of 100% (95% CI: 94.7–100). In contrast, the NSA-LFK showed a sensitivity of 59.3% (95% CI: 46.0–71.3) and a specificity of 97.1% (95% CI: 90.0–99.2). Pooled analysis showed that sensitivity was significantly higher for SA-LFK than for NSA-LFK in the Mild + Moderate group (77.8% vs. 59.3%, *p* = 0.004), whereas in the Severe group, sensitivity was higher for SA-LFK (97.4% vs. 89.7%) but the difference was not statistically significant (*p* = 0.25).

### 3.6. Adenoviral Load by Clinical Severity

To investigate the correlation between adenoviral DNA copy number and clinical severity, viral loads were compared among the severity groups. A Kruskal–Wallis test followed by Dunn’s multiple comparison test revealed no statistically significant differences in adenoviral DNA copy number among the mild, moderate, and severe groups (all adjusted *p* > 0.05).

**Figure 3:**
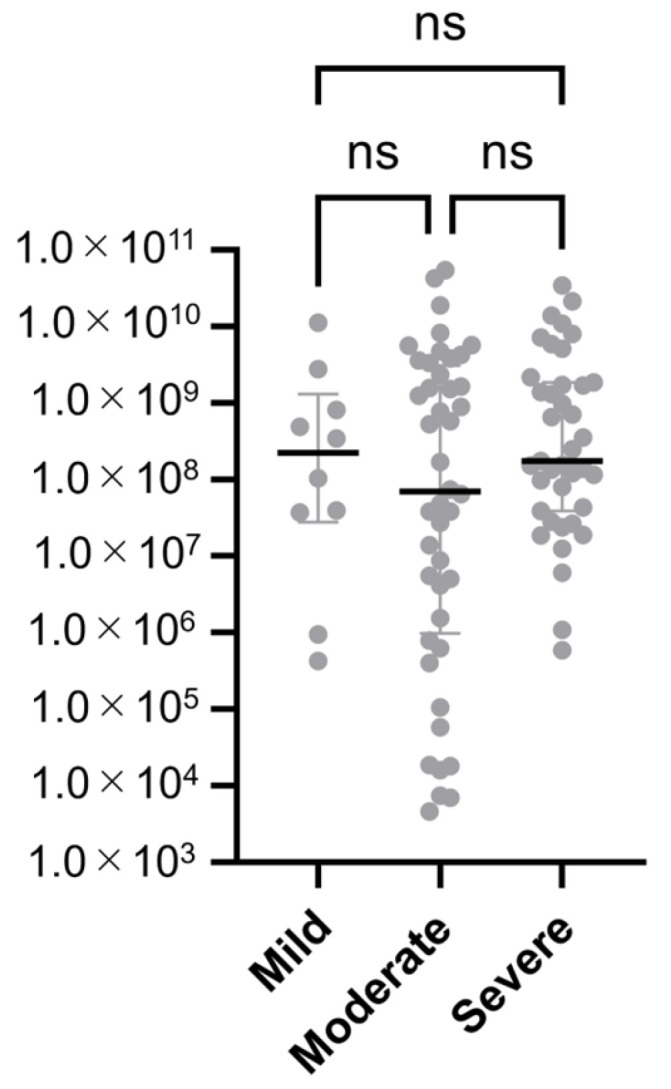
Adenoviral DNA copy numbers by clinical severity. Each gray dot represents an individual sample, and the horizontal bars indicate the median values with interquartile ranges. “ns” denotes non-significant differences among the mild, moderate, and severe groups (Kruskal–Wallis test followed by Dunn’s post hoc analysis; all adjusted *p* > 0.05).

As shown in Figure 3, adenoviral DNA copy numbers were plotted for each severity group using a logarithmic scale. Statistical comparisons between groups indicated no statistically significant differences in viral load.

These findings suggest that clinical severity, as assessed by anatomical extent of conjunctival inflammation, does not necessarily reflect the viral load present in tear fluid.

## 4. Discussion

### 4.1. Overview of Study Findings

This multicenter, prospective study provides the first comprehensive clinical evaluation of SA-LFKs for adenoviral conjunctivitis across diverse clinical settings. Using qPCR as the reference standard, SA-LFK consistently outperformed NSA-LFK in both sensitivity and specificity.

As summarized in Table 2, SA-LFK achieved 86.0% sensitivity and 100% specificity, while NSA-LFK showed lower sensitivity (72.0%) and slightly reduced specificity (98.1%). The difference in sensitivity was statistically significant (McNemar’s test, *p* < 0.001). These findings support the enhanced diagnostic accuracy of silver amplification [5], particularly in cases with low viral loads or mild conjunctival symptoms.

Quantitative analysis using qPCR revealed that the SA-LFK maintained high positivity rates even at viral loads as low as 10^5^ copies/test, whereas the NSA-LFK showed a marked decline below 10^6^ copies/test.

Although diagnostic sensitivity varied by clinical severity—with the SA-LFK detecting approximately 80% of mild and moderate cases and the NSA-LFK detecting approximately 60%—statistical analysis showed no significant differences in adenoviral DNA copy number among the mild, moderate, and severe groups. This may be attributable to the nature of PCR, which quantifies viral DNA rather than antigenic burden or host inflammatory response, potentially explaining the lack of correlation between severity and viral load.

Although we expected a trend of increasing viral load and sensitivity from mild to severe cases, our data did not support this assumption. Copy number showed no significant differences, and sensitivity was highest in severe cases but similar between the mild and moderate groups. These findings suggest that factors other than viral load, such as anatomical distribution or host response, may influence both clinical severity and kit performance.

Genotyping Via hexon, fiber, and penton region PCR identified eight AdV types, including types 2, 3, 37, 53, 54, 56, 64, and 85. SA-LFKs showed higher positivity rates across most genotypes, particularly for types 3, 37, and 85. In particular, cases caused by types 2 [17] and 85 [13] exhibited clear clinical features of epidemic keratoconjunctivitis (EKC), as previously reported, and were confirmed to have typical EKC presentations.

Notably, the sensitivity for type 53 was relatively lower. Previous studies have shown that types 53 and 56 tend to cause milder symptoms and shorter disease durations compared with types 8, 37, and 54 [1]. This variation in clinical severity may explain the reduced detection sensitivity for type 53, as milder symptoms are often linked to lower viral loads [4]. However, as our results showed no significant differences in viral load across severity groups, it is likely that kit performance characteristics, rather than viral quantity, play a more critical role in detection sensitivity.

Furthermore, among the 200 patients included in this study, preauricular lymphadenopathy, a classic sign of EKC, was observed in 30. Of these, 70% (21/30) tested positive using the SA-LFK, while 53.3% (16/30) tested positive using the NSA-LFK.

Importantly, when EKC is clinically suspected based on findings such as follicular conjunctivitis and lymphadenopathy [18], clinicians should interpret negative LF kit results with caution. In such cases, clinical judgment should be based on a comprehensive assessment that considers not only the test kit results but also the patient’s symptoms and prevailing epidemiological conditions [4].

Although hexon nested PCR [10] was useful for genotyping, its complexity and contamination risk limit its routine clinical use. In contrast, the SYBR Green-based qPCR method used in this study was practical and reliable. A cutoff value of 4.6 copies/μL was established to minimize false positives, validated through melt curve analysis and control testing.

qPCR provided consistent and informative results, making it a definitive reference standard. Its ability to apply system-specific cutoff values makes it particularly well-suited for evaluating lateral flow assays, where precise quantification is essential for correlating viral load with detection sensitivity.

In addition, the recent clinical availability of iodine-based ophthalmic disinfectants in Japan for the treatment of EKC [19,20] has increased the importance of early and accurate AdV diagnosis. As these antiviral interventions are most effective when administered during the early stages of infection, the ability of SA-LFK to detect mild and moderate cases with high sensitivity is clinically relevant.

### 4.2. Interpretation and Broader Context

Our findings align with previous reports indicating that silver amplification enhances the sensitivity of lateral flow assays, particularly in respiratory and ocular viral infections [6,21]. The ability of SA-LFKs to detect adenoviral conjunctivitis even at lower viral loads supports the hypothesis that signal amplification can compensate for the limited antigen presence in early or mild disease stages. This is particularly relevant in outpatient settings, where rapid diagnosis is essential for infection control.

### 4.3. Clinical and Public Health Implications

Given the global burden of adenoviral conjunctivitis and its potential for nosocomial outbreaks [22], the implementation of highly sensitive point-of-care diagnostics such as SA-LFKs may contribute to improved triage, reduced transmission, and more targeted use of antiviral agents. The robust performance across genotypes also suggests applicability in regions with diverse adenovirus circulation patterns.

### 4.4. Future Research Directions

Further studies are warranted to validate these findings in larger and more diverse populations. Longitudinal studies assessing viral load dynamics and clinical outcomes over time would help clarify the relationship between severity and viral burden.

### 4.5. Limitations

This study has several limitations. First, all four clinical sites were located in Japan, which may limit the generalizability of the findings to other populations and healthcare systems. Second, the total sample size (*n* = 200), particularly the small number of mild cases (*n* = 14), may reduce the statistical robustness of severity-based analyses. The small number of mild cases, the evaluation of clinical severity at a single time point (initial visit), and the absence of adjustment for interobserver variability may have affected the accuracy of severity assessment. Additionally, clinical severity was assessed only at the initial visit, which may affect the accuracy of severity classification and its correlation with viral load. Although PCR quantifies viral DNA, it may not fully reflect antigenic burden or host immune response, potentially explaining the lack of correlation between severity and viral load.

Third, the findings are specific to the two evaluated LF kits and may not be generalizable to other SA-LFK or NSA-LFK platforms. Inter-observer agreement for severity grading was not assessed, and κ statistics were not calculated. The order of LF testing was not randomized, although equal aliquots of tear fluid were used to minimize depletion bias.

Finally, analytical performance parameters such as limit of blank (LoB), limit of detection (LoD), linearity, and intra/inter-run coefficient of variation (CV) were not evaluated. Although the SA-LFK showed no false positives and the NSA-LFK showed two false positives against qPCR, potential cross-reactivity with other ocular pathogens (e.g., herpes simplex virus, enterovirus, and common bacteria) cannot be excluded. A dedicated cross-reactivity panel was not implemented and should be considered in future studies.

### 4.6. Conclusions

This multicenter prospective study demonstrates that silver-amplified lateral flow kits (SA-LFKs) significantly improve diagnostic sensitivity for adenoviral conjunctivitis compared with non-amplified kits (NSA-LFKs), while maintaining excellent specificity. The superior performance of SA-LFKs was consistent across multiple adenovirus genotypes and was particularly evident in mild and moderate cases, where early detection is critical for infection control and timely therapeutic intervention.

Importantly, our findings revealed no significant correlation between viral load and clinical severity, suggesting that factors beyond viral quantity—such as anatomical distribution or host response—may influence detection performance. These insights underscore the value of SA-LFKs in real-world settings, especially where rapid and reliable diagnosis is essential.

Given the increasing availability of antiviral ophthalmic agents and the persistent risk of nosocomial outbreaks, the adoption of SA-LFKs in routine clinical practice may facilitate more effective containment strategies. Future large-scale and longitudinal studies are warranted to validate these findings across diverse populations and to further explore the relationship between viral dynamics, clinical severity, and diagnostic performance.

## Figures and Tables

**Table 1:** Clinical evaluation of SA-LFK and NSA-LFK kits using qPCR as the gold standard.

	SA-LFK Kit			NSA-LFK Kit			
	Positive	Negative		Positive	Negative		Total
qPCR-Positive	80	13		67	26		93
qPCR-Negative	0	107		2	105		107
Total	80	120		69	131		

**Table 2:** Diagnostic performance metrics of SA-LFK and NSA-LFK kits.

Kit	Sensitivity (%)	Sensitivity 95% CI	Specificity (%)	Specificity 95% CI	PPV (%)	PPV 95% CI	NPV (%)	NPV 95% CI	False Negative Rate (%)	False Positive Rate (%)
SA-LFK	86.0	77.5–91.7	100.0	96.5–100.0	100.0	95.4–100.0	89.2	82.3–93.6	14.0	0.0
NSA-LFK	72.0	62.2–80.2	98.1	93.4–99.7	97.1	90.0–99.5	80.2	72.5–86.1	28.0	1.9

**Table 3:** Diagnostic performance of SA-LFK and NSA-LFK kits by clinical severity.

Severity	Kit	Sensitivity (%)	Sensitivity 95% CI	Specificity (%)	Specificity 95% CI	PPV (%)	PPV 95% CI	NPV (%)	NPV 95% CI	False Negative Rate (%)	False Positive Rate (%)
Severe	SA-LFK	97.4	86.8–99.9	100	90.8–100.0	100	90.8–100.0	97.4	86.8–99.9	2.6	0
Severe	NSA-LFK	89.7	76.4–95.9	100	90.8–100.0	100	90.1–100.0	90.5	77.9–96.2	10.3	0
Mild + Moderate	SA-LFK	77.8	65.1–86.8	100	94.7–100.0	100	91.6–100.0	85.2	75.9–91.3	22.2	0
Mild + Moderate	NSA-LFK	59.3	46.0–71.3	97.1	90.0–99.5	94.1	80.9–99.0	75.3	65.4–83.1	40.7	2.9

## Data Availability

An anonymized minimal dataset (ID, severity, genotype, qPCR copies/mL, SA/NSA results) will be deposited in a public repository upon acceptance; the link and DOI will be provided in the final version. Supplementary Table S1 provides detailed case-level data.

## Supplementary Materials

The following supporting information can be downloaded at: https://www.mdpi.com/article/10.3390/v17111442/s1. Supplementary Table S1: Clinical and laboratory data of patients with adenoviral conjunctivitis included in this study. The table summarizes specimen information, clinical symptoms, severity of conjunctivitis, associated findings (e.g., lymph node swelling, conjunctival hemorrhage, corneal opacity), treatment details, and PCR results. Quantitative PCR (qPCR) copy numbers and accession numbers of reference sequences used for typing are also provided.

## Abbreviations

The following abbreviations are used in this manuscript:

AdV	Adenovirus
BLAST	Basic Local Alignment Search Tool
EKC	Epidemic Keratoconjunctivitis
LF	Lateral Flow
SA-LFK	Silver-Amplified Lateral Flow Kits
NSA-LFKs	Non-Silver-Amplified Lateral Flow Kits
PPV	Positive Predictive Value
NPV	Negative Predictive Value
qPCR	Quantitative Real-Time PCR

## References

[1] Aoki K., Gonzalez G., Hinokuma R., Yawata N., Tsutsumi M., Ohno S., Kitaichi N. (2019). Assessment of clinical signs associated with adenoviral epidemic keratoconjunctivitis cases in southern Japan between 2011 and 2014. Diagn. Microbiol. Infect. Dis..

[2] Ghebremedhin B. (2014). Human adenovirus: Viral pathogen with increasing importance. Eur. J. Microbiol. Immunol..

[3] Hage E., Espelage W., Eckmanns T., Lamson D.M., Panto L., Ganzenmueller T., Heim A. (2017). Molecular phylogeny of a novel human adenovirus type 8 strain causing a prolonged, multi-state keratoconjunctivitis epidemic in Germany. Sci. Rep..

[4] Kaneko H., Maruko I., Iida T., Ohguchi T., Aoki K., Ohno S., Suzutani T. (2008). The possibility of human adenovirus detection from the conjunctiva in asymptomatic cases during nosocomial infection. Cornea.

[5] Fujimoto T., Hanaoka N., Konagaya M., Kobayashi M., Nakagawa H., Hatano H., Tsukahara-Kawamura T., Uchio E., Kaneko H. (2019). Evaluation of a silver-amplified immunochromatography kit for adenoviral conjunctivitis. J. Med. Virol..

[6] Migita H., Ueno T., Tsukahara-Kawamura T., Saeki Y., Hanaoka N., Fujimoto T., Uchio E. (2019). Evaluation of adenovirus amplified detection of immunochromatographic test using tears including conjunctival exudate in patients with adenoviral keratoconjunctivitis. Graefes Arch. Clin. Exp. Ophthalmol..

[7] Matsushima Y., Nakajima E., Ishikawa M., Kano A., Komane A., Fujimoto T., Hanaoka N., Okabe N., Shimizu H. (2014). Construction of new primer sets for corresponding to genetic evolution of human adenoviruses in major capsid genes through frequent recombination. Jpn. J. Infect. Dis..

[8] Walsh M.P., Chintakuntlawar A., Robinson C.M., Madisch I., Harrach B., Hudson N.R., Schnurr D., Heim A., Chodosh J., Seto D. (2009). Evidence of molecular evolution driven by recombination events influencing tropism in a novel human adenovirus that causes epidemic keratoconjunctivitis. PLoS ONE.

[9] Park A., Lee C., Lee J.Y. (2024). Genomic Evolution and Recombination Dynamics of Human Adenovirus D Species: Insights from Comprehensive Bioinformatic Analysis. J. Microbiol..

[10] Okada M., Ogawa T., Kubonoya H., Yoshizumi H., Shinozaki K. (2007). Detection and sequence-based typing of human adenoviruses using sensitive universal primer sets for the hexon gene. Arch. Virol..

[11] Fujimoto T., Matsushima Y., Shimizu H., Ishimaru Y., Kano A., Nakajima E., Adhikary A.K., Hanaoka N., Okabe N. (2012). A molecular epidemiologic study of human adenovirus type 8 isolates causing epidemic keratoconjunctivitis in Kawasaki City, Japan in 2011. Jpn. J. Infect. Dis..

[12] Banik U., Adhikary A.K., Suzuki E., Inada T., Okabe N. (2005). Multiplex PCR assay for rapid identification of oculopathogenic adenoviruses by amplification of the fiber and hexon genes. J. Clin. Microbiol..

[13] Kaneko H., Hanaoka N., Konagaya M., Kobayashi M., Nakagawa H., Hatano H., Ikuta K., Sekiryu T., Fujimoto T. (2020). Five Cases of Epidemic Keratoconjunctivitis Due to Human Adenovirus Type 85 in Fukushima, Japan. Jpn. J. Infect. Dis..

[14] Darougar S., Grey R.H., Thaker U., McSwiggan D.A. (1983). Clinical and epidemiological features of adenovirus keratoconjunctivitis in London. Br. J. Ophthalmol..

[15] Aoki K., Kaneko H., Kitaichi N., Ohguchi T., Tagawa Y., Ohno S. (2011). Clinical features of adenoviral conjunctivitis at the early stage of infection. Jpn. J. Ophthalmol..

[16] Fujimoto T., Okafuji T., Okafuji T., Ito M., Nukuzuma S., Chikahira M., Nishio O. (2004). Evaluation of a bedside immunochromatographic test for detection of adenovirus in respiratory samples, by comparison to virus isolation, PCR, and real-time PCR. J. Clin. Microbiol..

[17] Kaneko H., Hanaoka N., Konagaya M., Tsukahara-Kawamura T., Kobayashi M., Nakagawa H., Hatano H., Ikuta K., Fujimoto T. (2019). Conjunctivitis Due to the Human Adenovirus Type 2 Variant Identified during Epidemic Keratoconjunctivitis Surveillance in Japan. Jpn. J. Infect. Dis..

[18] Dumitrache M., Dumitrache M. (2024). Ophthalmological Pathology of the Eye: Conjunctiva. Clinical Ophthalmology, A Guide to Diagnosis and Treatment.

[19] Tsukahara-Kawamura T., Hanaoka N., Uchio E. (2024). Evaluation of anti-adenoviral effects of the polyvinyl alcohol iodine ophthalmic solution. Jpn. J. Ophthalmol..

[20] Labib B.A., Minhas B.K., Chigbu D.I. (2020). Management of Adenoviral Keratoconjunctivitis: Challenges and Solutions. Clin. Ophthalmol..

[21] Mitamura K., Shimizu H., Yamazaki M., Ichikawa M., Nagai K., Katada J., Wada A., Kawakami C., Sugaya N. (2013). Clinical evaluation of highly sensitive silver amplification immunochromatography systems for rapid diagnosis of influenza. J. Virol. Methods.

[22] Kuo I.C., Gower E.W. (2021). Cost Savings From a Policy to Diagnose and Prevent Transmission of Adenoviral Conjunctivitis in Employees of a Large Academic Medical Center. JAMA Ophthalmol..

